# Advancements in Clinical Utilization of Recombinant Human Collagen: An Extensive Review

**DOI:** 10.3390/life15040582

**Published:** 2025-04-01

**Authors:** Isaac Wong Kai Jie, Kar Wai Alvin Lee, Song Eun Yoon, Jong Keun Song, Lisa Kwin Wah Chan, Cheuk Hung Lee, Eunji Jeong, Jin-Hyun Kim, Kyu-Ho Yi

**Affiliations:** 1 The Artisan Clinic, 435 Orchard Road, #20-03, Singapore 238877, Singapore; drwong@artisanclinic.sg; 2 EverKeen Medical Centre, Hong Kong; 3 Brandnew Aesthetic Surgery Clinic, Seoul, Republic of Korea; 4 Pixelab Plastic Surgery Clinic, Seoul, Republic of Korea; 5 College of Medicine, Central Michigan University, Saginaw, MI, USA; 6 You & I Clinic, Seoul, Republic of Korea; 7 Division in Anatomy and Developmental Biology, Department of Oral Biology, Human Identification Research Institute, BK21 FOUR Project, Yonsei University College of Dentistry, 50-1 Yonsei-ro, Seodaemun-gu, Seoul 03722, Republic of Korea

**Keywords:** recombinant human collagen, tissue engineering, wound healing

## Abstract

Introduction: Recombinant human collagen, developed through advanced recombinant DNA technology, has emerged as a cutting-edge biomaterial with diverse applications in medicine. It addresses significant limitations of animal-derived collagens, such as immunogenicity and the risk of zoonotic diseases. Objective: This review evaluates the clinical applications, benefits, and challenges associated with recombinant human collagen, focusing on its potential to transform medical and surgical practices. Methods: A comprehensive search was conducted in MEDLINE, PubMed, and Ovid databases using keywords such as “Recombinant Human Collagen”, “Collagen-Based Biomaterials”, “Clinical Applications”, “Tissue Repair”, and “Wound Healing”. Relevant studies, including clinical trials and diagnostic applications, were analyzed and classified according to the Oxford Centre for Evidence-Based Medicine evidence hierarchy. Findings: Recombinant human collagen demonstrates superior mechanical properties and controlled degradation rates compared to traditional collagen sources. Clinical studies highlight its effectiveness in accelerating wound closure, promoting dermal regeneration, and minimizing scarring, making it particularly valuable in chronic wound management and surgical interventions. In tissue engineering, recombinant human collagen scaffolds have shown potential for regenerating cartilage, bone, and cardiovascular tissues by supporting cell proliferation, differentiation, and matrix deposition. Additionally, its adaptability for forming hydrogels and matrices enhances its suitability for drug delivery systems, enabling controlled and sustained release of therapeutic agents. Conclusion: Recombinant human collagen represents a transformative advancement in clinical practice, providing a safer and more effective alternative to traditional collagen sources. Its demonstrated success in wound healing, tissue engineering, and drug delivery highlights its potential to significantly improve patient outcomes. However, challenges such as high production costs, regulatory complexities, and long-term biocompatibility remain barriers to widespread clinical adoption. Further research and collaboration between biotechnology developers and regulatory authorities are essential to fully realize its clinical potential.

## 1. Introduction

Collagens represent a diverse family of extracellular matrix proteins, characterized by their unique triple-helical structure and critical roles in tissue architecture and cellular function [1]. These glycoproteins are predominantly classified into fibrillar (types I–III, V, XI) and non-fibrillar collagens, each serving specialized functions across various biological systems [2]. Type I collagen, the most abundant collagen in the human body, constitutes approximately 90% of the organic bone matrix and provides critical structural support in skin, tendons, and ligaments, forming highly organized, crosslinked fibrils that contribute to tissue tensile strength. Type II collagen, predominantly found in cartilaginous tissues, serves as a primary structural component in articular cartilage, intervertebral discs, and the eye’s vitreous humor, enabling significant resistance to compressive forces. Type III collagen is characterized by its association with dynamic and elastic tissues, including blood vessels, skin, and internal organs, playing a crucial role in early embryonic development and wound healing processes [3].

Recombinant human collagen represents a groundbreaking advancement in biomaterials, with significant potential for diverse medical applications. As the most abundant protein in the human body, collagen plays a critical role in maintaining the structural integrity of tissues [3,4,5,6,7]. Traditionally, collagen has been sourced from animal tissues and widely utilized in wound healing, tissue engineering, and regenerative medicine [1,8,9,10,11,12,13,14,15]. However, the use of animal-derived collagen poses notable challenges, including immunogenicity, the risk of zoonotic disease transmission, inconsistent quality, and limited supply [16,17,18,19,20,21,22].

The development of recombinant collagen marked a pivotal advancement in biomedical engineering, emerging from early protein engineering efforts in the late 1980s [23]. Researchers first successfully expressed collagen-like sequences in Escherichia coli in 1990, with subsequent breakthroughs in expression systems enabling more accurate protein folding. By the early 2000s, scientists produced fully human-like collagen molecules, overcoming previous limitations of immunogenicity and structural inconsistencies [24]. These technological advancements progressively expanded collagen’s potential applications from experimental research to practical clinical interventions in wound healing and regenerative medicine [25]. The evolution of recombinant collagen technology has been characterized by continuous refinement of production techniques and an increasing ability to engineer collagen with precise biochemical properties [1].

Recent advancements in biotechnology have facilitated the development of recombinant human collagen through recombinant DNA technology. This innovative approach enables precise control over collagen’s molecular structure, allowing for the synthesis of biomaterials that closely mimic native human tissue. Compared with animal-derived collagen, recombinant human collagen offers enhanced mechanical strength, controlled degradation rates, and superior bioactivity. These attributes position it as a promising candidate for clinical applications, with the potential to significantly improve patient outcomes.

The clinical applications of recombinant human collagen span a wide range of fields. It has demonstrated notable benefits in wound healing, where it accelerates tissue repair and reduces scarring. In tissue engineering, recombinant human collagen scaffolds effectively support cell proliferation, differentiation, and matrix deposition, contributing to the regeneration of cartilage, bone, and other tissues. Furthermore, its adaptability in forming hydrogels and other matrices enhances its utility in drug delivery systems, enabling controlled and sustained therapeutic agent release.

In this review, ‘bioactivity’ refers to the ability of recombinant human collagen to promote cellular processes such as adhesion, proliferation, and differentiation, which are crucial for tissue regeneration.

This review aims to provide a comprehensive analysis of recombinant human collagen, focusing on its unique properties, production methods, and current clinical applications. By examining preclinical and clinical studies, the review will explore its transformative potential in modern medicine. Despite its promise, challenges remain, including high production costs, regulatory hurdles, and long-term biocompatibility concerns.

The review will emphasize the importance of continued research and interdisciplinary collaboration to overcome these challenges. The future of recombinant human collagen holds the potential to revolutionize biomaterial applications, ushering in a new era of improved therapeutic outcomes and enhanced quality of life for patients worldwide.

### Fundamental Research

Recent advances in recombinant human collagen research have created significant opportunities across multiple medical applications. Fundamental studies exploring the structural and processing properties of this biomaterial have established its viability for clinical use. Cheng et al. [26] demonstrated that atomization parameters like nozzle size and pressure critically affect the molecular weight, secondary structure, and bioactivity of recombinant human collagen type III, directly impacting its effectiveness in tissue engineering applications. Building on these structural insights, Hua et al. [27] utilized X-ray crystallography to analyze collagen type III’s triple helix region, revealing molecular arrangements that enhance cell adhesion and migration—properties particularly valuable for dermal substitutes and tissue regeneration therapies.

The biological performance of recombinant human collagen has been extensively characterized across several investigations. Wang et al. [28] documented its ability to enhance cell adhesion, proliferation, migration, and differentiation, establishing its fundamental value in wound healing and tissue repair processes. These cellular effects translate to clinical applications, as demonstrated by Zhang et al. [29], who investigated recombinant human type III collagen’s therapeutic impact on atrophic scars through its influence on the p38 MAPK signaling pathway. Their combined in vitro experiments and clinical trials revealed improved fibroblast proliferation, increased collagen synthesis, and noticeable enhancements in scar texture and morphology.

The evolution of recombinant collagen technology spans three decades of research, comprehensively reviewed by Fertala et al. [21]. Their analysis details how genetic engineering advancements have revolutionized collagen production, offering benefits including improved safety, consistent quality, and customizable properties for applications ranging from tissue repair to wound healing. This technological progression has enabled the diverse applications examined by Cao et al. [3], who evaluated recombinant human collagen’s role across various tissue engineering domains including skin, bone, cartilage, and vascular applications. Their review highlighted its biocompatibility and effectiveness in promoting tissue regeneration through specialized scaffolds.

Despite these promising advances, regulatory considerations remain crucial for clinical translation. Liu et al. [30] addressed the unique regulatory challenges surrounding recombinant human collagen, emphasizing the need for updated frameworks that account for its distinctive properties while ensuring safety and efficacy in collagen-based medical devices. Collectively, these studies demonstrate recombinant human collagen’s growing importance in modern medicine and its potential to advance tissue engineering, improve wound healing outcomes, and expand therapeutic options while highlighting areas requiring further development.

Table 1 provides a concise summary of key studies and their findings, highlighting the potential of recombinant human collagen in fundamental research.

## 2. Dermatological Applications

Recombinant human collagen has emerged as a versatile biomaterial for diverse dermatological applications, demonstrating significant advantages in skin protection, tissue regeneration, and wound healing. The molecular basis for these applications lies in collagen’s fundamental role in maintaining dermal structural integrity, which becomes compromised during processes like photoaging. UV exposure accelerates collagen degradation through oxidative stress and inflammatory cascades, leading to characteristic skin aging manifestations. Innovative approaches to counteract these effects include topical antioxidants, peptides, and regenerative therapies utilizing stem cells and growth factors [31]. When applied topically, recombinant collagen formulations enhance native collagen deposition, improve skin elasticity, and reduce inflammatory markers associated with UV damage [32].

Skin regeneration represents a primary application domain where recombinant human collagen provides structural and biological support for cellular processes. Collagen-based biomaterials, including hydrogels and scaffolds, offer versatile platforms for tissue repair by supporting cell adhesion and proliferation. These materials can be further enhanced through incorporation of bioactive molecules to improve clinical outcomes [33]. Advanced tissue-engineered skin models integrate both cellular and acellular approaches with sophisticated 3D culture systems, creating realistic environments for studying regenerative processes and testing therapeutic interventions. These engineered constructs address both structural and functional requirements for effective healing [34]. The development of bioengineered human skin for regenerative medicine has been further validated through human–mouse models, which provide valuable preclinical data on biocompatibility, wound healing efficacy, and tissue integration. These approaches reduce complications associated with traditional skin grafts while advancing clinical applications through scaffold-based and stem cell-enhanced techniques [35].

Wound healing applications of recombinant human collagen span diverse clinical scenarios, from acute injuries to chronic, difficult-to-heal wounds. Injectable hydrogels combining recombinant collagen type III with chitosan demonstrate enhanced healing properties, including faster wound closure, improved collagen deposition, and increased angiogenesis in animal models. These formulations provide structural support while leveraging chitosan’s antibacterial and biocompatible properties to create optimal healing environments [36]. For specific conditions like sunburned skin, bioactive triple-helical recombinant collagen gels accelerate healing by maintaining collagen’s critical structure for biological activity. These specialized formulations demonstrate measurable improvements in inflammation reduction, collagen deposition, and re-epithelialization [37]. Oral ulcers represent another condition responsive to recombinant human collagen type III therapy, which promotes cell proliferation, migration, and collagen deposition, ultimately accelerating wound closure and restoring tissue structure [38].

Challenging wound environments, particularly diabetic wounds, benefit from responsive hydrogel systems incorporating recombinant collagen. These advanced formulations address the unique pathophysiology of diabetic wounds, including chronic inflammation and delayed healing, through spatiotemporal release of therapeutic components. Significant improvements in wound closure, angiogenesis, and tissue regeneration have been demonstrated in diabetic models, highlighting the potential for clinical translation of these technologies [39].

Innovation in drug delivery systems has expanded recombinant collagen’s utility in dermatological applications. Microneedle patches integrating recombinant human collagen enhance transdermal drug delivery by improving therapeutic absorption while minimizing skin irritation. These systems offer novel methods for delivering vaccines and biologics, with collagen-coated microneedles demonstrating enhanced structural integrity and biocompatibility that optimize drug diffusion and reduce inflammatory responses [40]. Further advancement of microneedle technology with recombinant collagen specifically targets chronic wound care, facilitating controlled therapeutic release and improving healing outcomes by creating optimized local environments for tissue regeneration [41].

**Figure 1:**
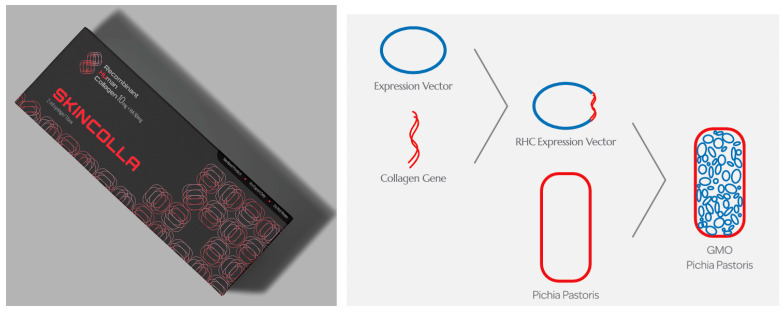
Human recombinant collagen is used as a skin booster and is injected intradermally to enhance skin quality. The product shown is SkinColla (Maypharm Inc., Seoul, Republic of Korea). It is 100% identical to human collagen, with its gene sequence matching the authenticated human collagen sequence in the GenBank database. The process includes gene design, gene cloning into a vector, production, analysis, and the extraction of human recombinant collagen [20].

Aesthetic dermatology represents an emerging application area for recombinant human collagen. Skin boosters enhance skin health and rejuvenation through advanced collagen supplementation [37]. For example, commercial products like SkinColla (Maypharm Inc., Seoul, Republic of Korea) improve skin quality by stimulating collagen synthesis and reducing the appearance of fine lines and wrinkles (Figure 1). These applications leverage recombinant collagen’s biocompatibility and structural properties to achieve both functional and aesthetic improvements in skin condition.

Collectively, these diverse applications demonstrate recombinant human collagen’s versatility and therapeutic potential across the dermatological field. By integrating this advanced biomaterial with innovative delivery systems and complementary technologies, significant improvements in treatment outcomes can be achieved for conditions ranging from photoaging to chronic wounds and aesthetic concerns.

Table 2 provides a concise summary of key studies and their findings, highlighting the potential of recombinant human collagen in dermatological applications.

## 3. Cardiovascular Applications

Recombinant human collagen type III (rhColIII) has emerged as a promising biomaterial for cardiovascular applications, offering significant advantages in both tissue engineering and medical device development. The controlled composition and improved safety profile of rhColIII compared with animal-derived alternatives make it particularly valuable for clinical translation. Vascular tissue engineering applications benefit from rhColIII’s ability to support essential cellular functions and provide structural integrity comparable to native tissue. Detailed investigations of the interaction between rhColIII and vascular endothelial cells demonstrate its capacity to positively influence cell proliferation and migration across various concentrations—critical processes for maintaining vascular integrity and promoting repair [42]. These fundamental cellular interactions, characterized through biochemical assays and advanced imaging techniques, form the foundation for developing functional vascular constructs and have led to advanced applications in blood-contacting medical devices where thrombosis and poor tissue integration traditionally present significant challenges.

The development of crosslinking strategies represents a critical advancement in rhColIII’s cardiovascular utility. These sophisticated techniques create stabilized acellular matrices while preserving the biomaterial’s inherent biological activity, striking a balance between structural durability and bioactivity. When applied to blood-contacting devices such as vascular grafts and heart valves, crosslinked rhColIII matrices demonstrate enhanced cell adhesion, proliferation, and endothelialization compared to conventional materials. The improved hemocompatibility of these materials, evidenced by reduced platelet activation and adhesion in detailed in vitro assays, addresses one of the primary limitations of cardiovascular implants by lowering thrombosis risk. In vivo performance confirms these benefits, with rhColIII-coated implants showing superior tissue integration and diminished inflammatory responses in carefully controlled animal models. The combined benefits of mechanical stability and biological functionality make crosslinked rhColIII particularly promising for complex cardiovascular applications requiring prolonged blood contact [43].

Cardiac regeneration represents another promising application domain, where injectable hydrogels responsive to the physiological environment of damaged cardiac tissue offer therapeutic potential following myocardial infarction. These advanced formulations, characterized by their tunable physical properties and biodegradation profiles, target multiple pathological processes simultaneously, including cardiomyocyte apoptosis, excessive inflammation, and insufficient angiogenesis. The controlled release capabilities of these systems, which respond to specific microenvironmental cues present in infarcted tissue, provide localized treatment while minimizing systemic effects—a crucial advantage for cardiac applications. Both in vitro studies with cardiac cell lines and in vivo evidence from animal models of myocardial infarction support the efficacy of these approaches. Comprehensive assessments of cardiac function, including echocardiography and hemodynamic measurements, demonstrate significant improvements in ventricular function, reduced scar tissue formation, and enhanced vascularization following treatment with these responsive hydrogel systems [32].

The surface modification of cardiovascular devices has been revolutionized through the incorporation of rhColIII in biomimetic coatings. By combining rhColIII with other bioactive molecules like hyaluronic acid through precisely controlled stepwise assembly techniques, researchers have developed extracellular matrix-mimetic surfaces that significantly improve device–tissue interactions. The structural characteristics of these coatings, including thickness, composition, and mechanical properties, have been optimized to maximize biological performance while maintaining the underlying device functionality. These sophisticated surface modifications enhance cellular responses including adhesion, proliferation, and migration while reducing inflammatory responses in vivo, as confirmed through both cell culture experiments and animal implantation studies. Such modifications represent a significant advancement in stent technology and hold promise for improving long-term outcomes of cardiovascular interventions by reducing restenosis and promoting functional endothelialization [44].

The ongoing evolution of rhColIII applications in cardiovascular medicine reflects broader trends in biomaterial development and personalized medicine. Interdisciplinary approaches integrating materials science, engineering, and medicine continue to drive innovation in this field, with particular emphasis on biodegradable materials that minimize complications associated with permanent implants. The development of smart devices incorporating sensors and drug delivery systems alongside rhColIII represents the cutting edge of this technology, offering possibilities for real-time monitoring and adaptive therapeutic responses. Recent advances in manufacturing technologies, including 3D printing and microfluidics, have further expanded the potential applications of rhColIII in patient-specific cardiovascular interventions. Despite these promising developments, regulatory hurdles remain significant barriers to clinical translation, requiring robust preclinical data and standardized manufacturing protocols. Current research aims to address these challenges through comprehensive toxicology studies and improved production methodologies. The collective evidence suggests that rhColIII has substantial potential to address persistent limitations in cardiovascular therapies through its versatile applications spanning tissue engineering to device modification [45]. Table 3 summarizes these key findings, highlighting the diverse cardiovascular applications of recombinant human collagen.

## 4. Gynecological Applications

Recent advancements in biomimetic extracellular matrices have shown significant promise in promoting endometrial regeneration, a critical area in gynecological therapeutics. Wei et al. [46] developed a composite hydrogel combining hyaluronic acid and recombinant human type III collagen. This innovative approach targets conditions such as infertility and endometrial injury by supporting natural repair processes. Hyaluronic acid is chosen for its biocompatibility and role in tissue hydration, while the recombinant collagen mimics the natural collagen found in the endometrium. Through in vitro experiments, the study evaluated the hydrogel’s effects on endometrial stromal cell proliferation, migration, and differentiation. In vivo studies in animal models further assessed its efficacy in promoting endometrial healing post-injury. Results indicated that the hydrogel significantly enhanced endometrial cell activities compared to controls, suggesting its potential as a therapeutic scaffold for uterine health (Level Ib).

In addressing vaginal atrophy, a condition prevalent among postmenopausal women characterized by vaginal dryness and thinning, researchers have explored non-hormonal alternatives. You et al. [47] focused on developing a biomaterial derived from recombinant human collagen type III, known for its excellent cell adhesion properties. This study highlights the limitations of traditional hormonal therapies and proposes a collagen-based solution to improve vaginal health. The researchers synthesized a biomaterial that retained high cell adhesion activity, optimizing its production to maintain structural integrity. Using a rat model of vaginal atrophy induced by ovariectomy, the study rigorously assessed the biomaterial’s impact on tissue morphology, cellular proliferation, and biochemical markers. The results demonstrated significant improvements in the vaginal environment, with increased epithelial thickness and enhanced fibroblast activation, effectively reversing signs of vaginal atrophy (Level IIb).

In the context of chronic endometritis, a persistent inflammatory condition of the endometrium, recombinant human collagen has been investigated for its immunomodulatory effects. You et al. [48] explored the role of the immune microenvironment, particularly macrophage behavior, in mediating inflammation and tissue repair. The study hypothesized that recombinant human collagen could modulate the immune response, shifting macrophage polarization from a pro-inflammatory (M1) to an anti-inflammatory (M2) phenotype. This shift was associated with reduced pro-inflammatory cytokine production and increased anti-inflammatory factors, promoting tissue regeneration. In animal models, the administration of recombinant collagen significantly improved histopathological features of the endometrium, reduced inflammatory cell infiltration, and enhanced tissue repair, highlighting its potential as an immunomodulatory agent (Level IIa).

Innovations in treating pelvic floor dysfunction have also emerged, with Li et al. [45] developing a novel injectable hydrogel derived from recombinant human collagen. Known for its biocompatibility and ability to promote cellular activities, this hydrogel was tested in a rat model to evaluate its effectiveness in reducing adverse tissue remodeling and enhancing pelvic floor function. The study detailed the synthesis of the hydrogel, emphasizing its high cell adhesion activity, crucial for facilitating cellular interactions and promoting healing. Preclinical evaluations included characterizing the hydrogel’s properties, such as viscosity and injectability, and its ability to support cell adhesion and proliferation. In vivo studies showed significant improvements in pelvic floor function and tissue architecture, with reduced adverse remodeling and increased collagen deposition, underscoring its promise for tissue engineering applications (Level IIa).

Table 4 provides a concise summary of these key studies, highlighting the potential of recombinant human collagen in various gynecological applications. While the current findings are based on animal models, translating these results to human trials requires comprehensive preclinical safety assessments and rigorous ethical considerations. Future research should focus on developing standardized protocols for human trials, addressing potential risks, obtaining necessary regulatory approvals, and establishing robust informed consent mechanisms.

## 5. Oncological Applications

Liu et al. [49] explore a novel application of recombinant humanized collagen type III in breast cancer treatment, contributing to the growing body of research on extracellular matrix interactions in cancer progression. The research focuses on the role of recombinant humanized type III collagen in modulating breast cancer cell behaviors, particularly targeting autophagy, proliferation, and migration. The authors hypothesize that recombinant humanized type III collagen possesses bioactive properties that can inhibit cancer cell activity, with a specific focus on discoidin domain receptor 1 (DDR1) signaling. This mechanism is consistent with emerging research demonstrating the complex role of DDR1 in cancer progression [50,51].

Similar collagen-based interventions have been observed in other cancer types, such as colorectal cancer, where collagen interactions demonstrate significant cellular regulatory potential [51]. Previous research by Payne et al. [52] in lung cancer and Zeng et al. [53] in ovarian cancer have similarly investigated collagen–receptor interactions, suggesting a potential broader applicability of this approach across different malignancies. These studies underscore the importance of exploring the generalizability of Liu et al.’s findings beyond breast cancer.

Experimental approaches, including cell viability assays, migration assays, and autophagy analysis, revealed significant inhibition of breast cancer cell proliferation and migration. The findings suggest that recombinant humanized type III collagen reduces autophagic activity, potentially interfering with cancer cell survival and metastatic potential.

## 6. Discussion

Recombinant human collagen has emerged as a pivotal biomaterial in the field of tissue engineering and regenerative medicine. Its diverse applications span several medical disciplines, offering promising outcomes in wound healing, scar treatment, tissue repair, and regenerative therapies.

### 6.1. Wound Healing and Skin Regeneration

One of the primary areas where recombinant human collagen has demonstrated significant clinical potential is in wound healing and skin regeneration. Studies have shown that recombinant human collagen formulations, such as injectable hydrogels and microneedle patches, effectively accelerate the healing of chronic wounds, including diabetic ulcers and burn injuries [39,54]. For instance, Xiong et al. [36] demonstrated that recombinant human collagen type III combined with chitosan in injectable hydrogels significantly improved wound healing due to their antibacterial and antioxidative properties. These hydrogels offer a moist environment conducive to healing, facilitate cell migration and proliferation, and can be tailored for sustained release of therapeutic agents.

Furthermore, Zhang et al. [29] found that recombinant human collagen type III significantly enhanced the healing process of atrophic scars by modulating the p38 MAPK signaling pathway. This indicates that recombinant human collagen not only supports the physical closure of wounds but also actively participates in the biochemical processes that underlie tissue repair and regeneration.

### 6.2. Treatment of Photoaging Skin

Photoaging, caused by chronic exposure to ultraviolet radiation, results in collagen degradation and skin damage. Recent advancements have shown that recombinant human collagen can mitigate these effects. Wang et al. [32] investigated the impact of recombinant human collagen on UV-induced photoaging in vivo and observed substantial improvements in skin integrity and appearance. The study suggested that recombinant human collagen’s regenerative properties help restore the skin’s collagen matrix, enhance hydration, and improve overall skin elasticity and resilience, making it a valuable therapeutic option for photoaging skin.

### 6.3. Oral Ulcer and Endometrial Regeneration

Recombinant human collagen has also shown promise in the treatment of oral ulcers and endometrial regeneration. Shuai et al. [38] reported that recombinant human collagen type III significantly promoted the healing of oral ulcers, demonstrating its potential in dental and oral health applications. This is particularly important as oral ulcers can cause significant discomfort and affect a patient’s quality of life.

In gynecology, recombinant human collagen’s application extends to endometrial regeneration. Wei et al. [46] developed a biomimetic extracellular matrix containing recombinant human collagen, which significantly enhanced endometrial regeneration. This finding is crucial for reproductive medicine, as it offers a potential solution for conditions like Asherman’s syndrome and chronic endometritis, where endometrial damage impairs fertility.

### 6.4. Cardiovascular and Oncology Applications

The versatility of recombinant human collagen is evident in its application in cardiovascular therapies. Yang et al. [44] highlighted the development of extracellular matrix-mimetic coatings for cardiovascular stents using recombinant human collagen, which showed improved biocompatibility and endothelialization. This innovation can potentially reduce the risk of stent-related complications, such as thrombosis and restenosis, thereby improving the outcomes of cardiovascular interventions.

In oncology, recombinant human collagen’s functional attributes are being explored for their antitumor effects. Liu et al. [49] demonstrated that recombinant human collagen type III exhibited high antitumor activity against breast cancer cells by inhibiting autophagy, proliferation, and migration through the DDR1 pathway. This points to a novel therapeutic strategy where recombinant human collagen not only provides structural support but also directly combats cancerous cells, opening new avenues for cancer treatment.

### 6.5. Regulatory and Production Perspectives

The production and regulatory landscape for recombinant human collagen-based medical devices has also evolved to keep pace with these innovations. Liu et al. [30] discussed the regulatory challenges and advancements in the development of recombinant collagen-based medical devices, emphasizing the importance of consistent quality and safety standards. The scalability and reproducibility of recombinant human collagen production have been enhanced through biotechnological advancements, such as the use of transgenic silkworms for collagen production [55].

### 6.6. Challenges and Future Directions

Despite the promising clinical applications, several challenges must be addressed to fully realize the potential of recombinant human collagen. These include optimizing the production processes to ensure cost-effectiveness and scalability, enhancing the stability and bioactivity of recombinant human collagen formulations, and conducting extensive clinical trials to validate efficacy and safety.

Future research should focus on exploring the synergistic effects of recombinant human collagen with other biomaterials and therapeutic agents. For example, combining recombinant human collagen with growth factors, cytokines, or stem cells could further enhance its regenerative capabilities. Additionally, the development of advanced delivery systems tailored for specific clinical needs will be crucial in broadening the applicability of recombinant human collagen.

Our review underscores that while recombinant human collagen offers superior mechanical properties and controlled degradation rates, significant challenges in its production pertain to the high costs of recombinant DNA technology, specialized equipment requirements, and the complexities of ensuring functional post-translational modifications. Addressing these production challenges is essential for enhancing scalability and reducing costs, thereby enabling broader clinical adoption and application.

Further investigation into the long-term effects and stability of recombinant human collagen is crucial for ensuring patient safety and material efficacy. Despite a reduced immunogenic response compared with animal-derived collagens, comprehensive assessments of long-term biocompatibility and potential adverse reactions remain pivotal. Additionally, developing robust formulations that maintain the structural integrity of recombinant human collagen under various environmental conditions during storage and transportation is necessary to meet stringent regulatory standards and ensure therapeutic effectiveness.

The utilization of recombinant DNA technology in producing human collagen also brings forth ethical and regulatory considerations. Ethical debates center on the implications of genetic manipulation, while regulatory challenges involve proving consistency and safety in the manufacturing processes. As advancements continue, interdisciplinary collaborations will be instrumental in navigating these ethical and regulatory landscapes, facilitating the development of innovative solutions that harness the full clinical potential of recombinant human collagen. Ongoing research and dialogue among scientists, regulators, and the community are crucial for developing and using recombinant human collagen responsibly, maximizing patient benefits and minimizing risks.

In assessing the long-term biocompatibility of recombinant human collagen, it is crucial to consider a range of factors that ensure patient safety and treatment efficacy. The potential risks associated with long-term use, such as immunogenic reactions and chronic inflammatory responses, require thorough examination. The immune response to these biomaterials can vary significantly, influenced by the degree of biomaterial purification and the presence of any residual non-human sequences. Additionally, the degradation of this collagen in the body impacts both the mechanical properties of the implant and the local tissue response. Ideally, the degradation products should be non-toxic and resorbable or eliminable by the body to avoid undesirable inflammatory reactions. Clinical outcomes documented in various studies have generally shown promising results in terms of efficacy and minimal adverse reactions. However, the need for ongoing monitoring and long-term studies is imperative to fully understand the safety profile of recombinant human collagen. Regulatory bodies play a crucial role in this process, requiring rigorous post-market surveillance to track the real-world performance of these biomaterials and ensure they meet long-term safety and effectiveness standards.

Beyond technical challenges, recombinant human collagen faces significant economic barriers, with production costs 10–20-fold higher than animal-derived alternatives [56]. This cost differential reflects sophisticated manufacturing requirements and impacts clinical accessibility, particularly in resource-limited situations. Regulatory hurdles further complicate translation, as these products occupy ambiguous classification boundaries between medical devices, biologics, and combination products [30]. Ramshaw (2016) highlighted the challenge of demonstrating equivalence to traditional collagen while validating unique advantages through costly clinical studies [15]. Ethical considerations include equitable access questions and transparent informed consent processes, especially regarding human-derived genetic templates used in production [57]. Addressing these multifaceted challenges requires collaborative approaches between stakeholders to establish frameworks that balance innovation, safety, accessibility, and ethical implementation.

## 7. Conclusions

Recombinant human collagen has proven to be a versatile and effective biomaterial with significant clinical applications in wound healing, skin regeneration, oral and endometrial repair, cardiovascular therapies, and oncology. Ongoing research and development efforts are key to overcoming existing challenges and unlocking the full therapeutic potential of recombinant human collagen. With continued advancements, recombinant human collagen is poised to become a cornerstone in regenerative medicine and biomedical applications.

## Figures and Tables

**Table 1:** Fundamental research and dermatological applications of recombinant human collagen.

References	Focus	Key Findings
[26]	Atomization effects on collagen III	Nozzle size/pressure alter molecular properties; enhanced tissue engineering suitability
[21]	30-year collagen research review	Production/safety advances; tailored properties for tissue repair and regeneration
[3]	Tissue engineering applications	Promote cell proliferation and regeneration in skin, bone, cartilage, and vascular tissues
[28,29]	Atrophic scars; cell behavior	p38 MAPK pathway activation; improved fibroblast activity and wound healing
[27]	Triple helix structural properties	Structure enables cell adhesion/migration for tissue regeneration applications
[30]	Regulatory challenges	Safety requirements and regulatory framework needs for collagen-based devices

**Table 2:** Dermatological applications of recombinant human collagen.

Reference	Research Focus	Key Findings
[31]	Collagen degradation and photoaging	UV-induced oxidative stress accelerates degradation; antioxidants and growth factors mitigate photoaging
[35]	Bioengineered human skin	Bioengineered skin using human–mouse models reduces complications and improves wound healing outcomes
[33]	Collagen-based biomaterials	Hydrogels/scaffolds enhanced with bioactive molecules improve tissue repair outcomes
[34]	Tissue-engineered skin models	Cellular/acellular approaches with 3D culture systems improve skin regeneration
[40]	Collagen-coated microneedle patches	Enhance drug absorption, minimize skin irritation, and optimize therapeutic diffusion
[36]	Injectable collagen–chitosan hydrogels	Promote wound closure, collagen deposition, and angiogenesis; antibacterial properties using collagen-coated microneedles
[41]	Microneedles with recombinant collagen	Enable controlled therapeutic release for chronic wounds; optimize local environment
[37]	Triple-helical collagen gels for sunburn	Reduce inflammation; improve collagen deposition and re-epithelialization
[38]	Recombinant collagen for oral ulcers	Accelerates wound closure, promotes cell proliferation and migration, and restores tissue structure
[39]	Responsive hydrogels for diabetic wounds	Improve wound closure, angiogenesis, and tissue regeneration using spatiotemporal collagen release
[32]	Recombinant collagen for UV damage	Enhances collagen deposition, skin elasticity; reduces inflammation markers as treatments for skin rejuvenation and repair

**Table 3:** Cardiovascular applications of recombinant human collagen.

Reference	Focus of Study	Methodology	Key Findings
[42]	Recombinant collagen III–endothelial cell interactions	Biochemical assays and cellular imaging techniques	Recombinant collagen positively influences endothelial cell proliferation and migration, supporting vascular repair
[43]	Crosslinked recombinant collagen III for blood-contacting devices	Hydrogel fabrication, hemocompatibility testing, and animal studies	Crosslinked matrices improve cell adhesion and endothelialization, and reduce thrombosis risk
[32]	Injectable hydrogels for cardiac regeneration	In vitro/in vivo studies on apoptosis reduction and angiogenesis	Responsive hydrogels improve cardiac function, reduce scarring, and promote vascularization
[44]	Cardiovascular stent modification with hyaluronic acid–collagen III	In vitro/in vivo studies on matrix-mimetic coatings	Matrix-mimetic coatings enhance cell adhesion and proliferation and reduce inflammation
[45]	Biomaterials for cardiovascular disease treatment	Systematic review of innovations in cardiovascular therapies	Biodegradable materials and smart devices improve outcomes and patient recovery

**Table 4:** The potential of recombinant human collagen in gynecological applications.

Reference	Focus Area	Methodology	Key Findings
[46]	Biomimetic matrix for endometrial regeneration	In vitro/in vivo studies on endometrial cell activities post-injury	Hyaluronic acid–collagen hydrogel promotes cell activities and enhances tissue regeneration
[47]	Recombinant collagen III for vaginal atrophy	Rat model of vaginal atrophy; assessed tissue morphology and cellular markers	Significant improvement in vaginal environment with increased epithelial thickness and fibroblast activation
[48]	Recombinant collagen for chronic endometritis	In vitro/in vivo approaches; evaluated macrophage polarization and immune microenvironment	Shift from M1 to M2 macrophage phenotype; reduced inflammatory cytokines and enhanced tissue repair
[45]	Injectable collagen hydrogel for pelvic floor dysfunction	Preclinical studies; evaluated hydrogel properties and efficacy in rat model	Improved pelvic floor function; reduced adverse tissue remodeling and enhanced collagen deposition

## Data Availability

No new data were created or analyzed in this study. Data sharing is not applicable to this article as no datasets were generated or used.
